# Neuropsychopharmacological profiling of scoparone in mice

**DOI:** 10.1038/s41598-021-04741-3

**Published:** 2022-01-17

**Authors:** Joanna Kowalczyk, Barbara Budzyńska, Łukasz Kurach, Daniele Pellegata, Nesrine S. El Sayed, Jürg Gertsch, Krystyna Skalicka-Woźniak

**Affiliations:** 1 Independent Laboratory of Behavioral Studies, Medical University of Lublin, Chodźki 4A, 20-093 Lublin, Poland; 2 Institute of Biochemistry and Molecular Medicine, University of Bern, Bühlstrasse 28, 3012 Bern, Switzerland; 3 Department of Pharmacology and Toxicology, Faculty of Pharmacy, Cairo University, Kasr El-Aini St., Cairo, 11562 Egypt; 4 Department of Natural Products Chemistry, Medical University of Lublin, Chodźki 1, 20-093 Lublin, Poland

**Keywords:** Secondary metabolism, Drug development

## Abstract

Scoparone (6,7-dimethoxycoumarin) is a simple coumarin from botanical drugs of Artemisia species used in Traditional Chinese Medicine and Génépi liquor. However, its bioavailability to the brain and potential central effects remain unexplored. We profiled the neuropharmacological effects of scoparone upon acute and subchronic intraperitoneal administration (2.5–25 mg/kg) in Swiss mice and determined its brain concentrations and its effects on the endocannabinoid system (ECS) and related lipids using LC–ESI–MS/MS. Scoparone showed no effect in the forced swimming test (FST) but, administered acutely, led to a bell-shaped anxiogenic-like behavior in the elevated plus-maze test and bell-shaped procognitive effects in the passive avoidance test when given subchronically and acutely. Scoparone rapidly but moderately accumulated in the brain (Cmax < 15 min) with an apparent first-order elimination (95% eliminated at 1 h). Acute scoparone administration (5 mg/kg) significantly increased brain arachidonic acid, prostaglandins, and *N*-acylethanolamines (NAEs) in the FST. Conversely, subchronic scoparone treatment (2.5 mg/kg) decreased NAEs and increased 2-arachidonoylglycerol. Scoparone differentially impacted ECS lipid remodeling in the brain independent of serine hydrolase modulation. Overall, the unexpectedly potent central effects of scoparone observed in mice could have toxicopharmacological implications for humans.

## Introduction

Coumarins are an independent class of shikimate-derived natural products with a 2H-chromen-2-one core scaffold. In profiling studies, various coumarins show a surprisingly broad range of biological activities in vitro and in vivo^1^. In addition, different coumarins, both synthetic and natural, have demonstrated effects on the central nervous system (CNS) in preclinical in vivo experiments^1,2^, suggesting their penetration through the blood–brain barrier (BBB). However, only a few studies have addressed the bioavailability and pharmacokinetics of natural coumarins in the brain, and there is a gap between the reported descriptive behavioral effects and the insights generated from bioanalytical measurements.

Scoparone (6,7-dimethoxycoumarin) is widely present in mugwort (also wormwood) plant species [*Artemisia species* (spp.)]. Regarding Traditional Chinese Medicine (TCM), the pharmacology of scoparone has been extensively studied in preclinical liver inflammation mouse models^3^. As non-terpenoid volatile, scoparone is present in essential oils^4^ and is a major natural product in Génépi liquor produced from *Artemisa spp*. plants, including *A. genepi*. Using high-performance countercurrent chromatography (HPCCC) we isolated scoparone as a major constituent from the extracts of the alpine wormwood species *Artemisia umbelliformis* Lam. (white genepì) used in the distillation of Génépi, a well-known traditional herbal liqueur or aperitif originating from the Alpine regions of Europe^5,6^. Although the bitter sesquiterpene lactones and the ketone monoterpene, thujone are the best-known psychoactive constituents of *Artemisia* spp.^6–8^, we hypothesized that scoparone might contribute to the alleged psychoactive or neurotoxic effects of mugwort-based alcoholic drinks^5,9^. To date, the potential pharmacological effects of scoparone in the CNS have not been studied. To our knowledge, the only report related to the central effects refers to pilocarpine and methylscopolamine-induced seizures in mice, where relatively high doses of scoparone (25–100 mg/kg) injected intraperitoneally (*i.p.*) attenuated the seizures' duration and decreased inflammatory processes in both the hippocampus and prefrontal cortex^10^. Although a recent comprehensive study on the rapid elimination of scoparone in humans and its metabolism in mouse, rat, pig, dog, and rabbit liver microsomes highlighted species-related differences in phase I metabolism^11^, the brain penetration and central effects of scoparone are unclear^12^.

**Figure 1:**
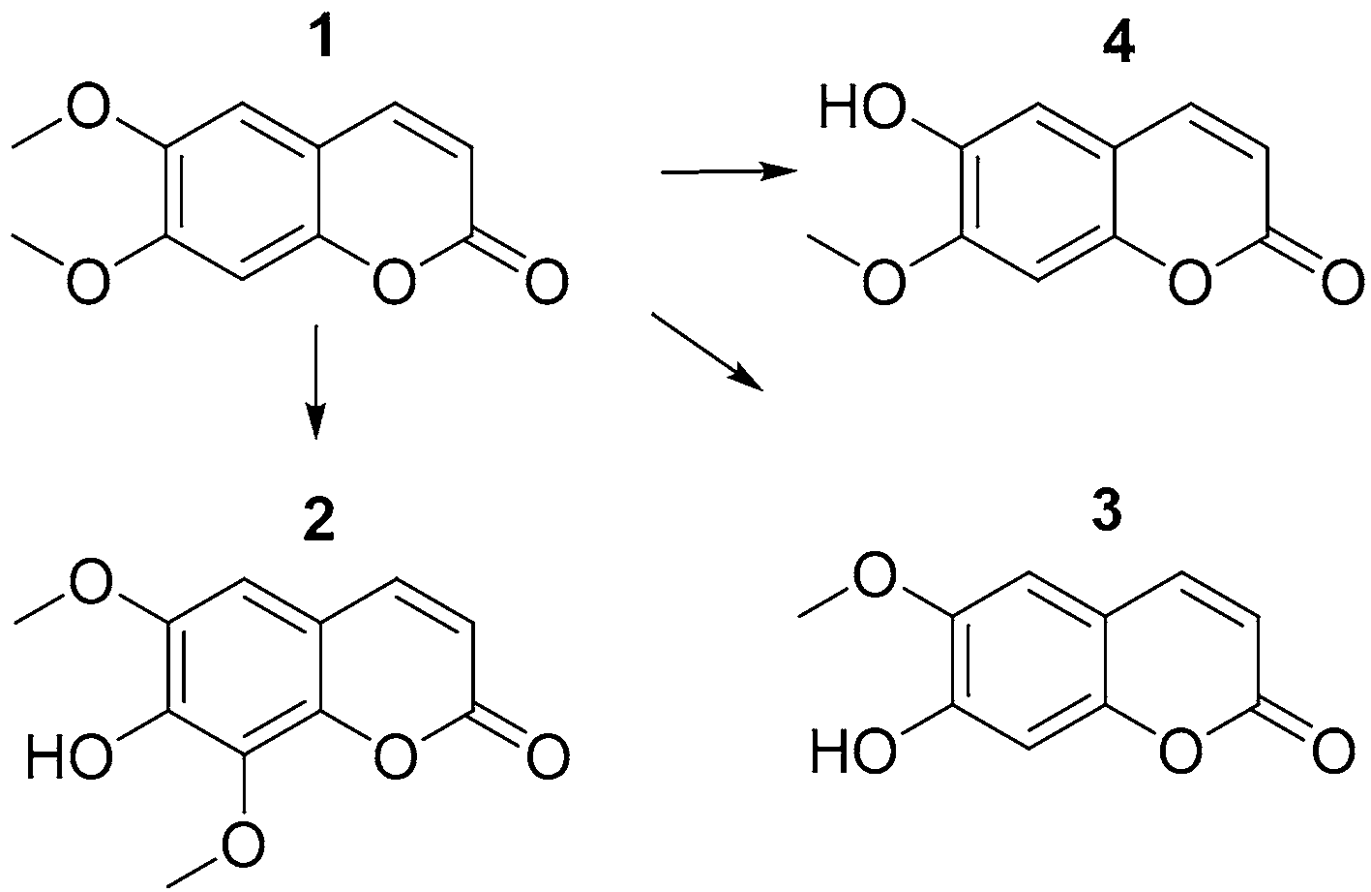
Chemical structures of scoparone (1) and its peripheral metabolites isofraxidin (2) scopoletin (3) and isoscopoletin (4).

In the present study, we specifically assessed the neuropharmacological effects of scoparone (Fig. 1) in a test battery of complex behavioral paradigms, including (a) depression-like behavior in the forced swim test (FST), (b) anxiety-like behavior in the elevated plus-maze (EPM), and (c) learning and memory in the passive avoidance test (PA) after acute and subchronic, (i.p.) injections of scoparone (2.5–25 mg/kg) in Swiss albino mice. In addition, the PA paradigm was applied to assess scoparone's procognitive/neuroprotective effects in both lipopolysaccharide (LPS) and scopolamine-induced mouse impaired learning and memory^13,14^.

We aimed to correlate the central effects with the concentration of scoparone and its major metabolites (Fig. 1) in mouse brain tissue when the compound was administered alone or in combination with borneol, a natural monoterpene shown to improve drug delivery to the brain^15^ possibly via p-glycoprotein inhibition^14^. It has already been shown that borneol promotes the accumulation of other drugs in brain tissue^15,16^. However, there is little evidence that it can enhance the brain permeability of coumarins. Thus, one of the aims was to check whether borneol increases the level of scoparone in the CNS. This is relevant because there is already a well-established TCM pure natural preparation derived from plants and marketed in China promoting neurogenesis, where borneol is co-administered with herbal drugs from the Apiaceae family^17^. Using LC–MS/MS-based targeted lipidomics, we measured the effects of scoparone on the brain lipidome associated with the endocannabinoid system (ECS), a major lipid signaling network that regulates complex behaviors and stress responses in neuropsychiatric disorders^13^.

## Results

### Biphasic anxiogenic effect of acute treatment with scoparone in the EPM assay in male mice

**Figure 2:**
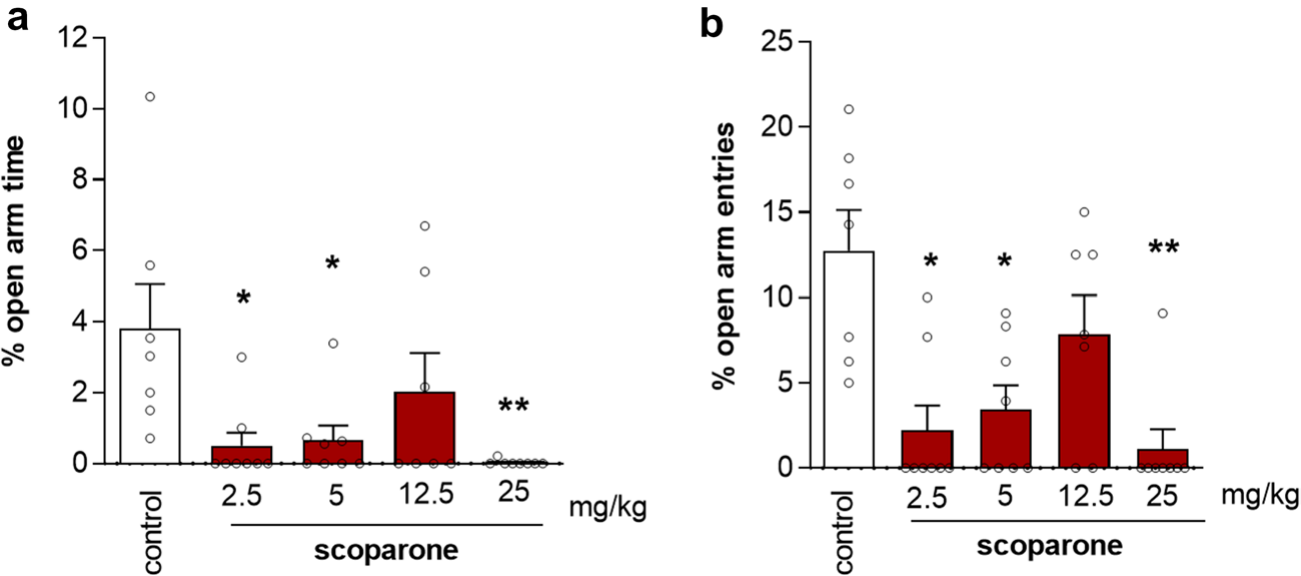
Scoparone induces anxiogenic effects. Dose-dependent (bell-shaped) effect of scoparone administration on anxiety-like behavior in Swiss albino male mice in the elevated plus maze (EPM) test. Figures show mean values ± SEM of the percentage time spent on the open arms (**a**) and the percentage of open arm entries (**b**) measured 30 min after an acute injection of scoparone (2.5, 5, 12.5, 25 mg/kg, i.p.) or vehicle; n = 8–9; *p < 0.05, **p < 0.01, vs. vehicle-treated control group, Tukey's test.

As shown in Fig. 2, acute administration of scoparone influenced the time spent on the open arms (one-way ANOVA: [F (4, 38) = 4.183; *p* = 0.0078]) (Fig. 2a) as well as the percentage of open arm entries (one-way ANOVA: [F (4, 38) = 4.514; *p* = 0.0057] (Fig. 2b).

A post-hoc analysis showed that scoparone at the doses of 2.5, 5 (*p* < 0.05), and 25 mg/kg (*p* < 0.01) significantly decreased the percentage of the time spent on the open arms as well as the percentage of open arm entries, indicating an anxiety-like behavior. Therefore, scoparone exhibited a noteworthy biphasic nonlinear bell-shaped dose–response effect with anxiogenic-like effects observed at the lower and higher doses. Conversely, upon subchronic treatment (daily injections for 14 days), scoparone did not show any effect in the EPM at the doses 2.5, 5, 12.5, and 25 mg/kg (Supplementary Table S4).

### Scoparone improved learning or memory acquisition in male mice in PA

**Figure 3:**
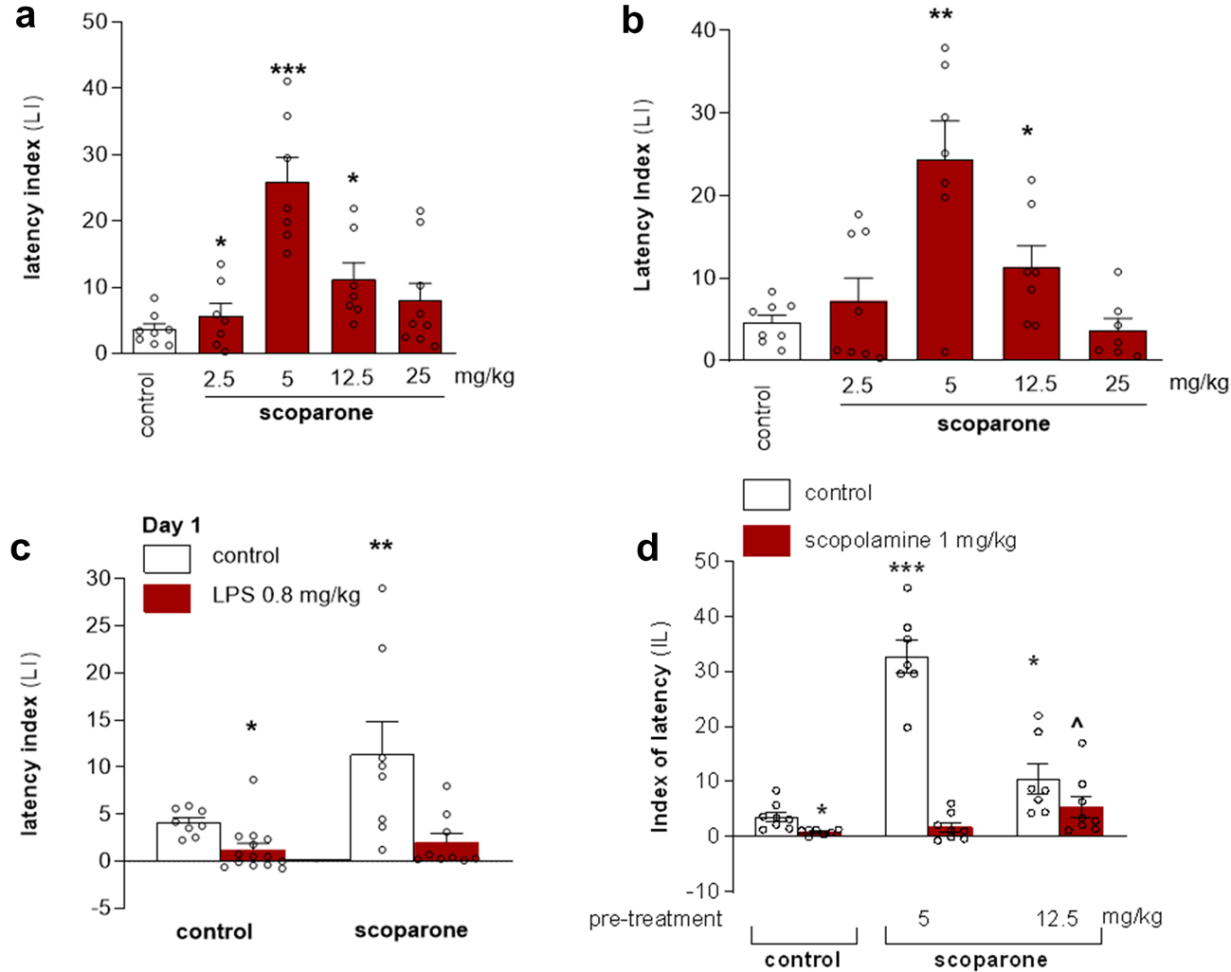
Scoparone shows procognitive effects in male Swiss albino mice the passive avoidance (PA) test. (**a**) Effects of an acute scoparone administration on the latency index (LI) during the acquisition trial using the PA test. Scoparone (2.5, 5, 12.5, 25 mg/kg; i.p.) or vehicle were injected 30 min before the first trial and mice were re-tested 24 h later; n = 8–9; the means ± SEM; *p < 0.05, ***p < 0.001 vs. vehicle-treated control group; Tukey’s test. (**b**) Effects of subchronic scoparone administration on the latency index (LI) during the acquisition trial using the PA test in mice. Scoparone (2.5, 5, 12.5 and 25 mg/kg; i.p.) or vehicle were administered for the six days. On the seventh day scoparone was administered 30 min before the first trial and mice were re-tested 24 h later; n = 8–9; the means ± SEM; *p < 0.05; **p < 0.01 vs. vehicle-treated control group; Tukey’s test. (**c**) Effects of subchronic administration of scoparone (15 mg/kg, i.p.) on LPS (0.8 mg/kg)-induced impairment of memory acquisition trial using the PA test in mice. Mice were injected with scoparone 60 min after LPS administration and then consecutively for 6 days. On the seventh day, scoparone was injected 30 min before the first trial, and animals were retested 24 h after the last injection; n = 8–9; the means ± SEM; *p < 0.05, **p < 0.01 vs. vehicle-treated control group; Bonferroni’s test. (**d**) Effects of acute administration of scoparone on scopolamine-induced impairment of memory acquisition trial using the PA test in mice. Scoparone (5 and 12.5 mg/kg, i.p.) was administered 30 min and scopolamine (1 mg/kg, i.p.) 20 min before the first trial and animals were re-tested 24 h after the last injection; n = 8–9; the means ± SEM; *p < 0.05; ***p < 0.001 vs. vehicle-treated control group, ^*p* < 0.05, vs. scopolamine-treated group; Bonferroni’s post hoc test.

Scoparone was also tested in the PA paradigm to assess its effect on learning and memory (Fig. 3). A single injection of scoparone (i.p.) had a statistically significant effect on latency index (LI) values for memory acquisition in the PA test (F (4, 33) = 7.486; *p* = 0.0002) (Fig. 3a). The post-hoc Tukey’s test confirmed that the treatment with scoparone at the doses 2.5, 5, and 12.5 mg/kg significantly increased LI values in male mice compared to those in the saline-treated control group (*p* < 0.05, *p* < 0.001, *p* < 0.05, respectively) (Fig. 3a), indicating that scoparone at these used doses improved learning or memory acquisition. No effect was observed when scoparone was delivered at dose of 25 mg/kg. Thus, scoparone exhibited nonlinear dose–response effects showing no significant effects at the higher doses. Since this effect was noticed after a single injection of scoparone**,** we decided to evaluate the effects of subchronic scoparone treatment (Fig. 3b). One-way ANOVA analysis showed that subchronic (14-days) administration of scoparone (2.5, 5, 12.5, and 25 mg/kg, i.p.) had a statistically significant effect on LI values for memory acquisition in the PA test (F (4, 37) = 0.0074; *p* = 0.0021). The post-hoc Tukey’s test confirmed that scoparone treatment significantly increased LI values in mice compared to those in the saline-treated control group (*p* < 0.01 for the dose of 5 mg/kg, and p < 0.05 for the dose of 12.5 mg/kg). Upon either acute or subchronic administration of scoparone, the dose of 5 mg/kg exerted the most pronounced effect (Fig. 3a,b). Noteworthy, a bell-shaped dose–response was recorded in the PA test after both acute and subchronic scoparone treatment, similar to those observed in the EPM (see above).

### Subchronic administration of scoparone did not improve learning and memory impairment by a single LPS injection in male mice in PA test

Because of the procognitive effects of scoparone observed in the PA, we next assessed whether scoparone could attenuate or protect against inflammation-mediated learning and memory impairment. Figure 3c shows the effect of subchronic scoparone treatment (15 mg/kg, i.p., for 7 days) on memory acquisition impaired by a single injection of LPS (0.8 mg/kg) during the retention trial in the PA task (two-way ANOVA: LPS pre-treatment [F (1, 35) = 10.14; *p* = 0.0032] and scoparone treatment [F (1, 35) = 6.72; *p* = 0.0141] showing a lack of protective effect [F (1, 35) = 4.47; *p* = 0.0522). The post hoc Bonferroni’s test confirmed that scoparone given subchronically significantly increased the LI value (*p* < 0.01). Since scoparone did not attenuate the LPS-induced amnesic effect, we concluded that this natural product does not protect from inflammation-related memory impairment.

### Acute scoparone administration prevented memory impairment induced by a single scopolamine injection in male mice in PA test

We next assessed whether scoparone could attenuate the memory deficits induced by scopolamine injection in the PA test. As expected, we observed a statistically significant memory impairment caused by scopolamine (1 mg/kg) treatment (F (1, 44) = 65.27;* p* < 0.0001). A significant effect of memory improvement (F (2, 44) = 25.91;* p* < 0.0001) and an interaction between scoparone and scopolamine (F (2, 44) = 33.61;* p* < 0.0001) was observed. The post-hoc Bonferroni’s test confirmed that scopolamine at the dose of 1 mg/kg significantly decreased LI values in mice in the PA test in comparison to the vehicle-treated mice, which confirms the amnesic effect of this drug (*p* < 0.05). It was also confirmed that scoparone, at the doses of 5 and 12.5 mg/kg, improved acquisition of memory and learning in this assay (*p* < 0.001, *p* < 0.05, respectively). Additionally, scoparone (12.5 mg/kg) prevented the amnesic-like effect of scopolamine (*p* < 0.05) as compared to scopolamine-treated mice (Fig. 3d). Because low and high scoparone doses (2.5 and 25 mg/kg) were inactive in the PA test, we did not consider these doses in the scopolamine memory impairment model.

### Effects of a single injection of scoparone on the levels of AChE, BChE, and FRAP in scopolamine-treated male mice

**Figure 4:**
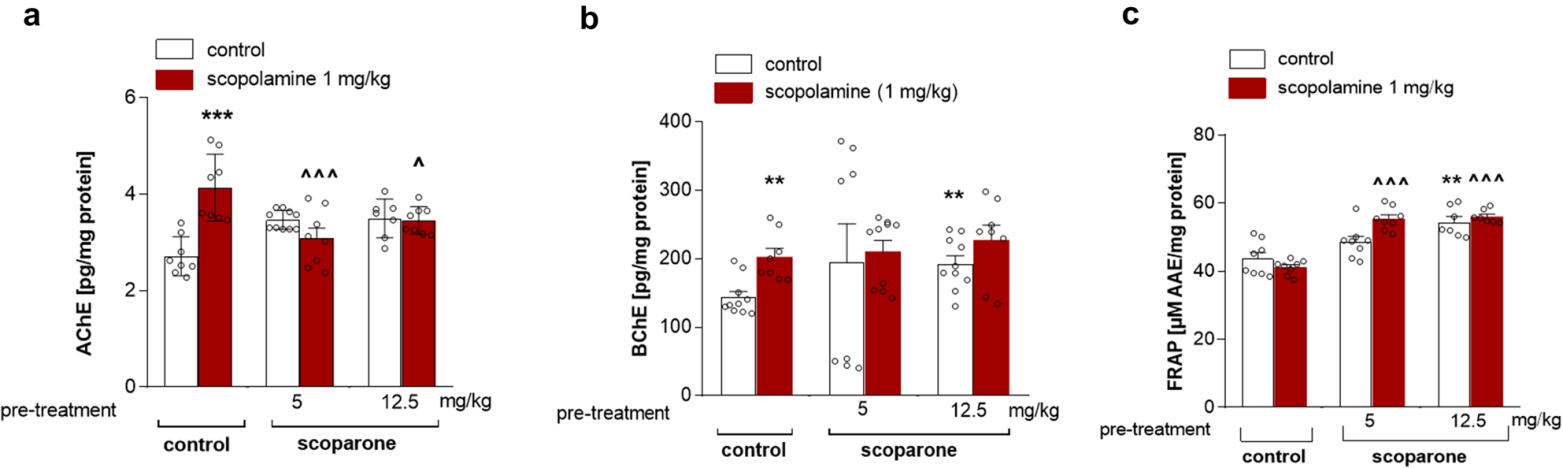
(**a**) Effects of acute administration of scoparone (5 and 12.5 mg/kg) on AChE brain levels induced by scopolamine administration in mice. Scoparone was administered 30 min and scopolamine (1 mg/kg, i.p.) 20 min before the first trial and animals were re-tested 24 h after the last injection; n = 8–9; the means ± SEM; ***p < 0.001 vs. vehicle-treated control group, ^*p* < 0.05, ^^^*p* < 0.001 vs. scopolamine-treated control group; Bonferroni’s test. (**b**) Effects of acute administration of scoparone on BChE level induced by scopolamine in mouse brain. Scoparone was administered 30 min and scopolamine (1 mg/kg, i.p.) 20 min before the first trial, and animals were re-tested 24 h after the last injection; n = 8–9; the means ± SEM; **p < 0.01 vs. vehicle-treated control group; Bonferroni’s test. (**c**) Effects of acute administration of scoparone on FRAP levels in mouse brain. Scoparone was administered 30 min and scopolamine (1 mg/kg, i.p.) 20 min before the first trial and animals were re-tested 24 h after the last injection, n = 8–9; the means ± SEM; **p < 0.01 vs. vehicle-treated control group, ^^^*p* < 0.001 vs. scopolamine-treated control group; Bonferroni’s test.

Because of the apparently protective effects of scoparone in the scopolamine PA test, in which the cholinergic system plays a primary role, we further assessed the effects on AChE and BChE levels in brain as well as antioxidative potential using the FRAP assay. Figure 4a shows the effects of acute scoparone treatment (5 and 12.5 mg/kg) administered alone or in combination with acute scopolamine injection (1 mg/kg) on AChE brain levels (scopolamine treatment (F (1, 50) = 4.65,* p* = 0.0387), scoparone pretreatment (F (2, 50) = 0.27,* p* = 0.7633) and scoparone-scopolamine interactions (F (1, 50) = 11.01, *p* = 0.0002); two-way ANOVA). The Bonferroni’s post hoc test showed a significant increase of the AChE concentration after single injection of scopolamine (*p* < 0.001) while no changes were noticed after single injection of scoparone. We observed that scoparone decreased the level of AChE in scopolamine injected mice (5 mg/kg,* p* < 0.001; 12.5 mg/kg,* p* < 0.05) in comparison with scopolamine-treated group (Fig. 4a). Figure 4b shows the effects of acute administration of scoparone (5 and 12.5 mg/kg) alone or in combination with acute injection of scopolamine on BChE level measured in the brain (scoparone pretreatment (F (2, 53) = 2.14,* p* = 0.1099), scoparone-scopolamine interactions effect (F (1, 53) = 0.67,* p* = 0.5178), scoparone treatment (F (1, 53) = 4.12,* p* = 0.0482); two-way ANOVA). The Bonferroni’s post hoc test confirmed the significant increase of the BChE concentration after single injection of scopolamine (*p* < 0.01). Figure 4c shows the effect of acute scoparone treatment (5 and 12.5 mg/kg), administered alone or in combination with an acute injection of scopolamine, on FRAP levels measured in brain homogenates (scoparone pretreatment (F (2, 45) = 33.82,* p* ≤ 0.0001)), scoparone–scopolamine interactions effect (F (1, 45) = 3.53,* p* = 0.0413), scopolamine treatment (F (1, 45) = 2.19,* p* = 0.1485); two-way ANOVA). The Bonferroni’s post hoc test showed a significant increase of the FRAP concentration after a single injection of scoparone at 12.5 mg/kg (*p* < 0.01). We observed an increased FRAP levels in the mice treated with scoparone (5 and 12.5 mg/kg) and scopolamine in comparison with scopolamine-treated mice (*p* < 0.001) (Fig. 4c).

### Concentrations of scoparone in the brain upon single i.p. injection and elimination kinetics in male mice

**Figure 5:**
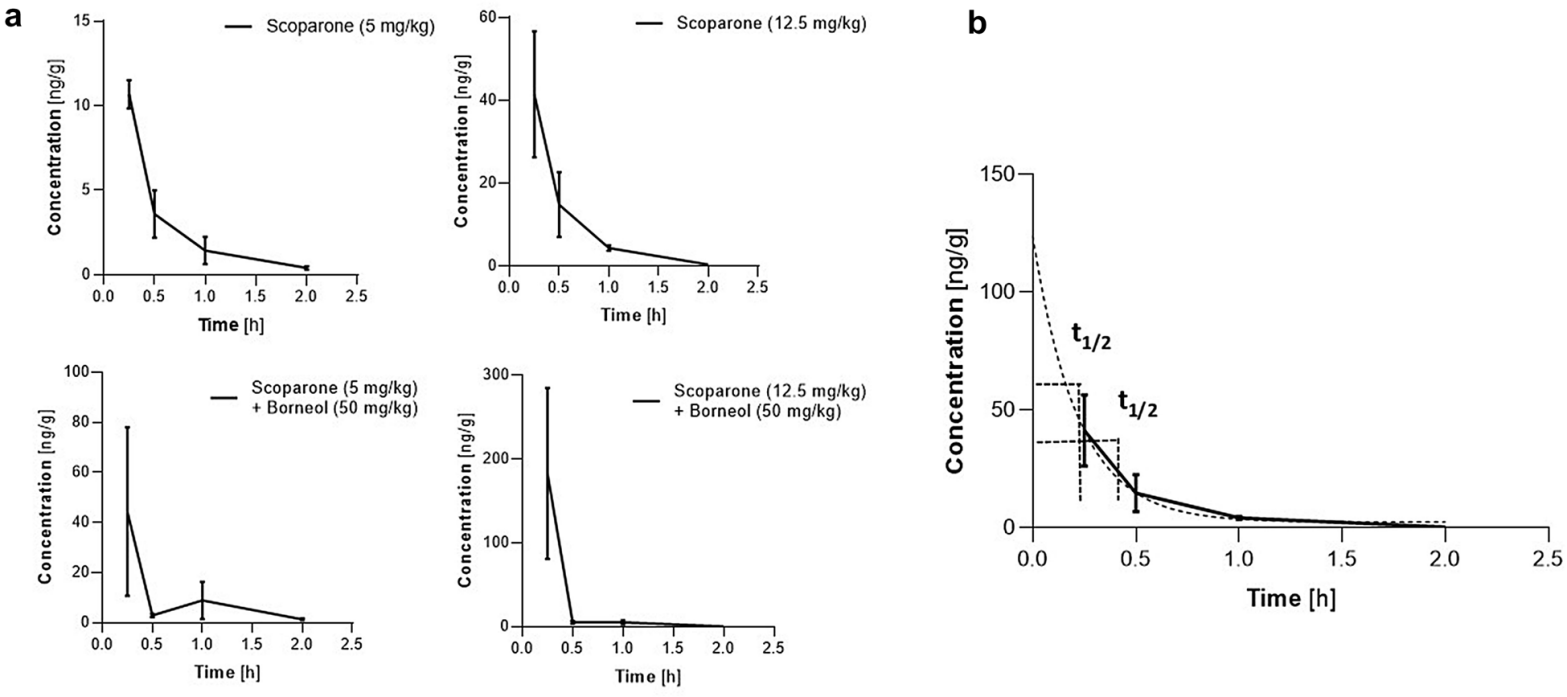
Pharmacokinetic analyses of scoparone in brain homogenates in Swiss albino male mice. (**a**) Time-dependent brain exposure upon administration of scoparone (i.p.) at 5 and 12.5 mg/kg and in combination with borneol (50 mg/kg) which significantly increased brain concentrations of scoparone. (**b**) Model of first order elimination of scoparone from mouse brain tissue upon administration of 12.5 mg/kg i.p. Data show mean values ± SD, n = 5.

We next quantified the concentrations of scoparone and its known metabolites (Fig. 5) in total mouse brain tissue using a quantitative LC–MS/MS method. Concentrations of scoparone [ng/g] were measured at different time points after acute i.p. injection of 5 mg/kg and 12.5 mg/kg (Fig. 5A). We did not detect scopoletin, isoscopoletin, and isofraxidin (limit of detection ≤ 0.2 ng/g brain tissue). Because the monoterpene borneol has been previously shown to increase brain concentrations of certain lipophilic natural products^15,16,18^, we performed an experiment, where both scoparone and borneol (50 mg/kg) were injected. Borneol was administered 60 min before scoparone (5 and 12.5 mg/kg, i.p.). The brain tissue was isolated 30 min after scoparone injection. As shown in Fig. 5A, borneol (50 mg/kg) increased the brain concentrations of scoparone by a factor 3. Interestingly, in this experiment we could also detect traces of isofraxidin in the brain, but only upon i.p. injection of 12.5 mg/kg scoparone in combination with 50 mg/kg borneol (data available on request). As summarized in Fig. 5B, scoparone was effectively eliminated from the mouse brain tissue showing an apparent first-order elimination kinetics. After 1 h, only traces of scoparone (2–6 ng/g brain tissue) could be detected under all conditions, independently on the C_max_ achieved.

### Targeted lipidomics shows differential modulation of endocannabinoids and NAEs by scoparone independent of serine hydrolase inhibition

**Figure 6:**
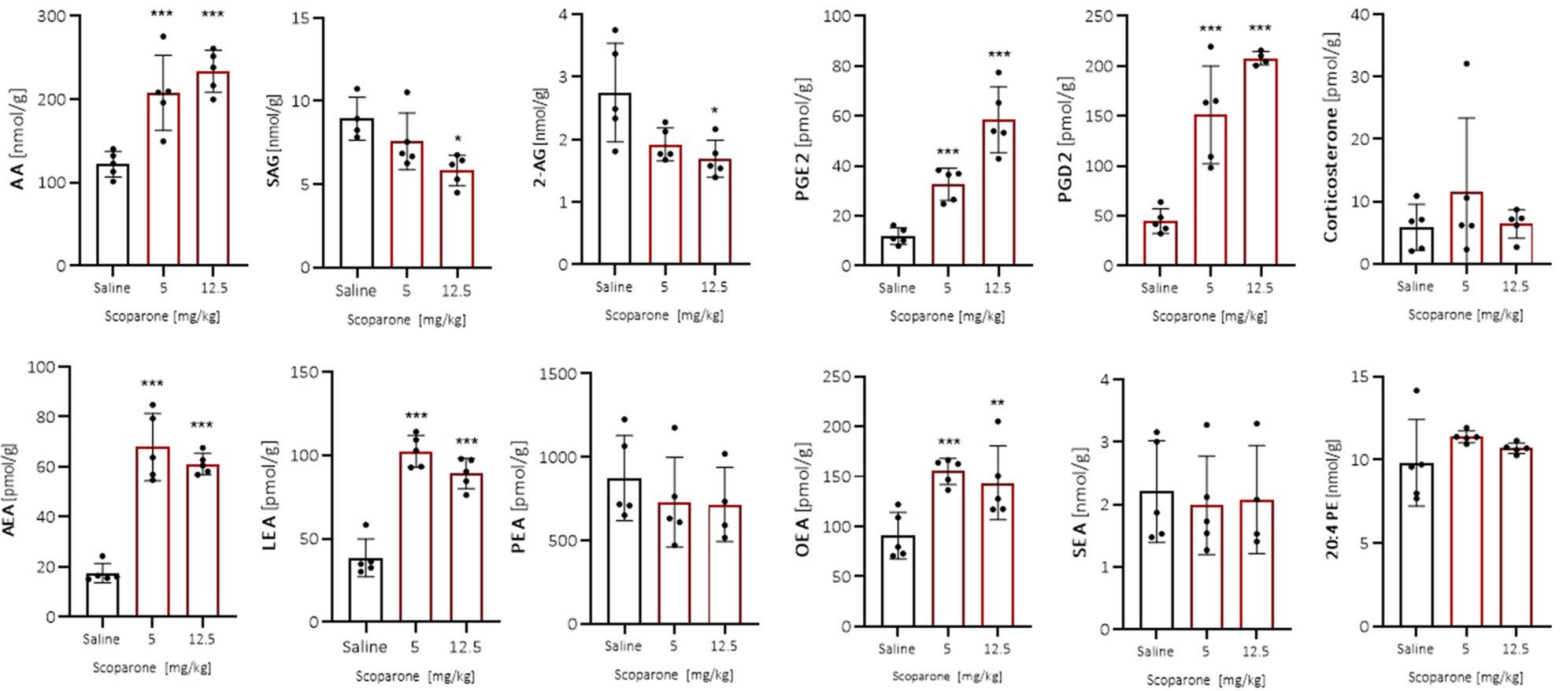
Acute scoparone treatment (5 and 12.5 mg/kg i.p.) on endocannabinoids and related lipids in the forced swim test (FST) in male Swiss albino mice. scoparone was injected 30 min before the FST and subsequently mice were sacrificed, and brains were obtained for measurement; n = 5; *p < 0.05; **p < 0.01; ***p < 0.001 vs. vehicle-treated control group; Tukey’s test. *AA* arachidonic acid, *SAG* 1-stearoyl-2-arachidonoyl-sn-glycerol, *2-AG* 2-arachidonoyl glycerol, *PGE2/PGD2* prostaglandins, *AEA* anandamide, *LEA* linoloyl ethanolamide, *PEA* palmitoyl ethanolamide, *OEA* oleoyl ethanolamide, *SEA* stearoyl ethanolamide, *20:4 PE* 1,2-diarachidonoyl-sn-glycero-3-phosphoethanolamine. Data show mean values ± SD, n = 5.

To get insights into possible mechanisms of action of scoparone, we performed targeted lipidomic analyses in mouse brain homogenates, focusing on the endocannabinoid system (ECS). Brains were collected both upon acute and subchronic administration of scoparone after the FST and EPM assays, respectively. Interestingly, we observed differential effects between the dosing regimens. Acute administration of scoparone led to a significant increase of AA and a trend to decrease 2-AG and its precursor diacylglycerol 1-stearoyl-2-arachidonoyl-sn-glycerol (SAG) at 12.5 mg/kg. The increased AA also resulted in a noteworthy significantly increased production of prostaglandins PGE2 and PGD2 (Fig. 6). Moreover, the levels of the endocannabinoid anandamide (AEA) and other *N*-acylethanolamines like linoloyl ethanolamide (LEA) and oleoyl ethanolamide (OEA) were increased significantly (Fig. 6). This was associated with a noteworthy trend towards increased 1,2-diarachidonoyl-sn-glycero-3-phosphoethanolamine (20:4 PE) which controls the production of *N*-arachidonoyl phosphatidylethanolagmine (NAPE) that is a precursor of AEA.

**Figure 7:**
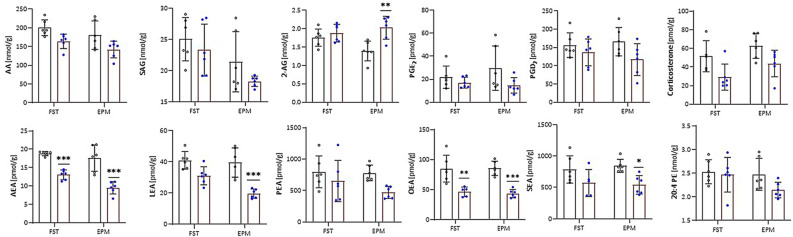
Effects of subchronic scoparone treatment (2.5 mg/kg, i.p., for 14 days) on endocannabinoids and related lipids in the forced swim test (FST) and elevated plus maze (EPM) paradigms in male Swiss albino mice. On day 15, scoparone was injected 30 min before the FST or EPM test and subsequently mice were sacrificed, and brains were obtained for measurements; n = 5; *p < 0.05; **p < 0.01; ***p < 0.001 vs. vehicle-treated control group. Bars with black dots show vehicle treated mice and bars with blue dots show scoparone treated mice. *AA* arachidonic acid, *SAG* 1-stearoyl-2-arachidonoyl-sn-glycerol, *2-AG* 2-arachidonoyl glycerol, *PGE2/PGD2* prostaglandins, *AEA* anandamide, *LEA* linoloyl ethanolamide, *PEA* palmitoyl ethanolamide, *OEA* oleoyl ethanolamide, *SEA* stearoyl ethanolamide, *20:4 PE* 1,2-diarachidonoyl-sn-glycero-3-phosphoethanolamine. Data show mean values ± SD, n = 5.

As shown in Fig. 7, subchronic scoparone treatment led to distinct effects as no modulation of AA or prostaglandins could be observed, but the opposite effects on NAEs which were significantly decreased. A trend towards decreased corticosterone levels could be seen. The strong decrease in AEA levels upon subchronic scoparone administration was most clearly visible in the EPM paradigm (Fig. 7).

**Figure 8:**
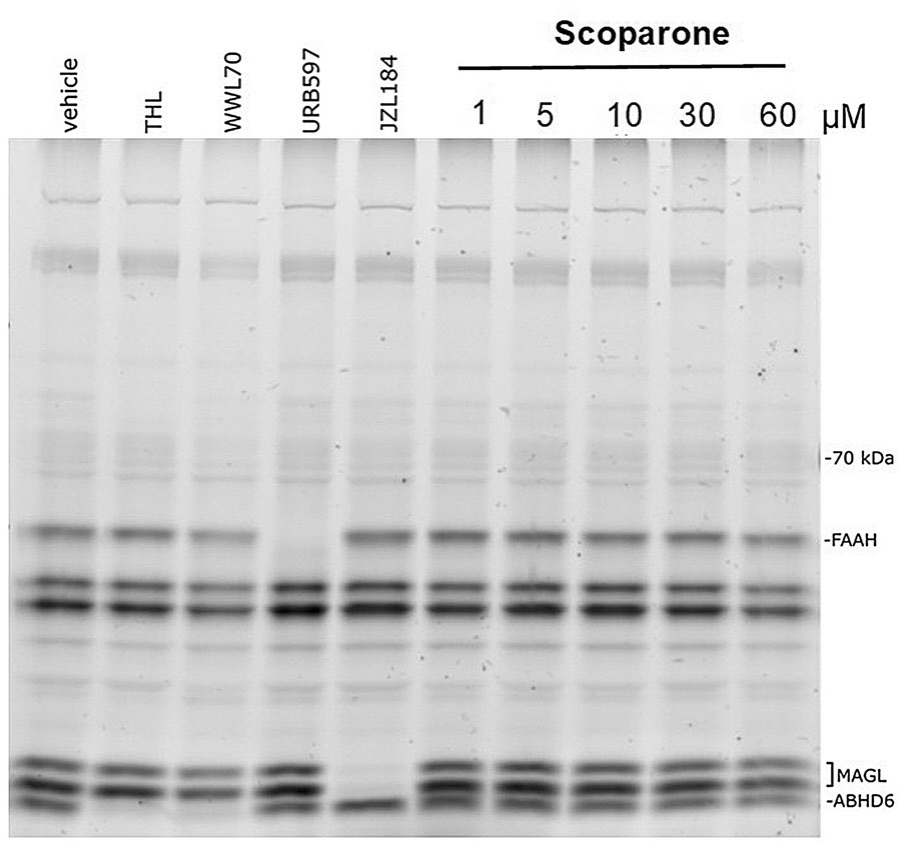
Activity-based protein profiling showing serine hydrolase activity in membranes from Swiss albino mouse brain tissues. Scoparone did not inhibit enzymes activity of serine hydrolases fatty acid amide hydrolase (FAAH), monoacylglycerol lipase (MAGL), and alpha–beta hydrolase 6 (ABHD6). DMSO was used as vehicle control. Specific inhibitors were used as positive controls for FAAH (URB597), MAGL (JZL184), ABHD6 (THL), and ABHD6/12 (WWL70). Image shows a representative blot of three independent experiments.

Intriguingly, in the subchronic scoparone treatment, the 2-AG levels were increased in the EPM, thus reflecting a change in endocannabinoid ratio compared to vehicle-treated mice. When comparing the saline-treated mice only (Figs. 6 and 7), we observed an increase of the levels of corticosterone (eightfold), AA (twofold), SAG (twofold), PGD2 (threefold), and 20:4 PE (threefold) in the saline controls between the acutely versus subchronically treated mice (FST), possibly reflecting the stress associated to daily injections. Also, SEA was increased (threefold) upon the repetitive injections, independent of the behavioral paradigm (FST vs. EPM). Overall, these data indicate behavior-specific effects of scoparone but an overall significant impact on the ECS and independent of corticosterone. The observed effects on endocannabinoids encouraged us to check whether scoparone directly inhibited the serine hydrolases involved in the differential degradation of endocannabinoids. As shown in Fig. 8, at concentrations up to 30 µM, scoparone did not influence serine hydrolases in brain homogenates as measured by activity-based protein profiling (ABPP) using a tetramethylrhodamine–labeled fluorophosphonate probe. In mouse brain tissue, MAGL was clearly active, together with FAAH and ABHD6. These enzymes showed characteristic molecular weights in the SDS PAGE and could be inhibited by selective inhibitors.

## Discussion

The apolar coumarin scoparone (6,7-dimethoxycoumarin) is mainly associated with the consumption of Génépi liquors that are very popular in the Alpine regions of Europe^6^. Distillation recipes of the French apéritif quina and some liquors like the Swedish-made Jeppson’s Malört use *Artemisia* species containing scoparone in Amaro. We hypothesized that scoparone might contribute to the psychoactive activity of liquors mentioned above and influence the brain and behavioral changes. We found that scoparone is a major coumarin in *Artemisia umbelliformis* Lam., a plant frequently used to distill mentioned above liquors, with an estimated amount of around 0.1–0.5% of the dried aerial parts.

For the peripheral effects, various mechanisms of action of scoparone have been proposed in vitro, mainly at concentrations in the micromolar concentration range^19–21^. However, these concentrations are not achieved in the brain. Thus, despite the wealth of data related to the peripheral anti-inflammatory and liver-protective effects of scoparone, it was hitherto unclear whether this coumarin also affects the brain. Here we show that scoparone exerts potent differential neuropharmacological effects in mice.

We used quantitative LC–MS/MS to measure scoparone in total brain homogenates at different time points after the FST and EPM assays. Because the administration of 5 or 12.5 mg/kg gave similarly low scoparone concentrations in the brain after 1 or 2 h (< 2 ng/g), respectively, we conclude that this coumarin is rapidly metabolized in the periphery, in agreement with previous literature. A fast metabolism of scoparone was described in the periphery after oral administration of herbal drugs^22^. After 30 min, around 0.1 µg/ml of scoparone was measured that further decreased to 0.01 µg/ml after 2 h. Overall, the bioavailability of coumarins was limited by liver metabolism by CYP1A2 enzymes that demethylate scoparone^23^. Scoparone was easily O-demethylated to scopoletin in the liver^24^, and different metabolites were generated^22^. Our study did not detect the accumulation of the major metabolites (Fig. 1) in the brain. However, after treatment with borneol and the highest dose of scoparone (12.5 mg/kg), traces of isofraxidin were detected. In the experiment in which borneol was co-administered with scoparone, no modulation of the scoparone-induced memory improvement was observed (Supplementary Fig. S2). Co-administration of 50 mg/kg of borneol, a monoterpene reported previously to specifically increase brain permeability of different drugs, hypothetically via *p*-glycoprotein inhibition^18^, increased scoparone concentrations in the brain by approximately 300%. The effect of borneol enantiomers on the pharmacokinetics of the simple coumarin osthole was previously investigated. Co-administration of these two compounds increases the bioavailability of osthole even by up 270%^25^. We found a similar acute administration of 12.5 mg/kg of scoparone yielded approximately 3–4 times the brain exposure of scoparone at 5 mg/kg. Our data thus suggest that scoparone could be eliminated from the brain via *p*-glycoprotein associated mechanisms. The concentration of scoparone (5 and 12.5 mg/kg) in the brain was highest at the earliest time point measured, after 15 min from the injection, and decreased in a time-dependent manner showing first-order kinetics.

Despite the low central bioavailability of scoparone the neuropharmacological profiling in different behavioral paradigms revealed an apparent effect specificity. Scoparone administered acutely (Supplementary Table S2) and subchronically (Supplementary Table S3) did not affect stress and depressive-like behaviors in the FST in both sexes, even though it exerted significant effects on endocannabinoids and related lipids (see below). We employed imipramine as a positive control in this model. Contrary to our expectations, we did not observe sex differences in behavior and acute drug action in the FST.

Upon acute administration of scoparone, in the EPM paradigm we observed anxiogenic-like effects in a bell-shaped curve of changes in the percentage of open arms time and entries (Fig. 2). Interestingly, the anxiogenic-like effect was not observed upon subchronic administration of scoparone (Supplementary Table S4). Unlike the acutely injected mice, these mice were more stressed (receiving repetitive daily injections) as judged by the corticosterone levels between vehicle control groups. Lipidomic analyses showed that subchronic administration of scoparone significantly reduced NAEs, including the endocannabinoid anandamide (AEA) reported to mediate anxiolytic effects in different preclinical models like the EPM^26^. The reversal of stress-induced anandamide deficiency is a postulated mechanism subserving the therapeutic effects of FAAH inhibition^27^.

Conversely, acute scoparone administration led to a very strong and unexpected increase of AA, prostaglandins, but also NAEs like anandamide and a concomitant decrease of 2-AG, which points towards a potent effect on lipid remodeling. 2-AG has been implicated in anxiety and is currently being studied in the context of stress-related conditions^13,28^. We have previously shown that endocannabinoid reuptake inhibitors lead to anxiolytic effects in the EPM via CB1 receptor activation, possibly mediated via 2-AG^29^. Thus, we speculate that the anxiogenic effect observed in the acute scoparone treatment could be related to the acute dysregulation of the arachidonate-endocannabinoid axis, i.e., resulting in increased prostaglandin and decreased 2-AG levels (see below). 2-AG deficiency has been implicated in anxiety and 2-AG can directly activate GABA-A receptors^30^, which mediates anxiolytic effects. Future studies will need to address this modulation in a brain region-specific manner, focussing on the amygdala, which is involved in anxiety-like behaviors in mice.

Dependent on the type of stress stimulus, PGE2 released from neurons, astrocytes, or microglia may trigger multiple neuronal pathways that result in behavioral stress responses including anxiety^31^. The observed effects on endocannabinoids are peculiar and suggest that scoparone significantly modulates the ECS in a behaviour context-dependent manner, a new finding in coumarin pharmacology. The inverse correlation between AA, prostaglandins, and 2-AG in the brain was first described by Nomura et al.^32^ and generally relates to changes in monoacylglycerol (MAGL) lipase expression. Thus, we measured whether MAGL or related serine hydrolases were a target of scoparone. Data obtained in ABPP experiments show that the serine hydrolases regulate endocannabinoid brain levels, namely MAGL, FAAH, and that ABHD6 were not inhibited by scoparone, and thus are not direct targets of this coumarin. While different natural products have been shown to interact with ECS targets^33^. Scoparone does not seem to exert a direct ECS modulation. Therefore, we will aim to elucidate the mode of action of scoparone in future studies.

Since scoparone did not modulate the FST behavior itself, we assume that this lipid remodeling was not causally involved in the FST behavior. Given the association of anandamide with depression-like behaviors in mice, this finding is unexpected because FAAH inhibitors are effective in the FST paradigm^34^.

The only emerging potentially beneficial effect was related to the PA test paradigm. It showed that scoparone injected both acutely and subchronically improved memory and learning. Because only male mice were tested in the PA assay, we currently do not know the procognitive effects in female mice, which is a limitation of this study. Interestingly, nootropics showing procognitive effects in the PA tests often also show anxiolytic-like effects^35^, in agreement with our observation in the EPM. To further explore the mechanisms of the procognitive effects of scoparone, we applied two different amnesia models related to scopolamine and LPS treatment, respectively. LPS as bacterial endotoxin causes acute systemic neuroinflammation and amyloidosis in vivo and triggers the production of pro-inflammatory cytokines^36^. In our study, scoparone did not reverse LPS-mediated cognitive impairment suggesting a lack of central anti-inflammatory effects. Acutely, we observed a significant increase of prostaglandins PGE2 and PGD2 in the FST, indicating at least temporary pro-inflammatory effects. Because timing issues hampered the experimental setup in the PA test for lipidomic analyses, we cannot be sure whether in all behavioral paradigms these lipids were modulated equally, which is a limitation of the study. However, the decrease of NAEs observed after subchronic scoparone treatment in the FST and EPM, if also present in the PA test, could limit the known anti-inflammatory action of these NAEs^37^.

On the other hand, the scopolamine model of dementia relates to cholinergic processes underlying memory functioning. Scopolamine was also found to cause lipid peroxidation by triggering free radical production, especially malondialdehyde (MDA) in combination with a decrease of antioxidative enzymes in the brain^38^. In our study, the scopolamine-induced memory impairments were attenuated by scoparone, indicating that scoparone treatment may also alter cholinergic neurotransmission. Biochemical data suggested that scoparone (5 and 12.5 mg/kg) inhibits the increase of AChE, but no BChE was observed upon scopolamine (1 mg/kg) injection. This is in line with our previous studies where the furanocoumarins xanthotoxin and bergapten diminished the level of AChE and increased the antioxidative capacity in the hippocampus and prefrontal cortex in the scopolamine model of dementia in mice^14,38^.

The antioxidant capacity was marginally but significantly higher in the scopolamine model after a single scoparone injection (5 and 12.5 mg/kg). Thus, in addition to effects on AChE, scoparone increased the antioxidative capacity in the brain that may, at least in part, underlie the procognitive effects.

The low concentration of 5 ng/g scoparone in brain tissue detected after 0.5 h after the injection of 5 mg/kg (i.p*.*) of scoparone was high enough to improve memory acquisition compared to the control group points towards a potent mode of action. Nevertheless, the Cmax before the behavioral assay was estimated to be higher than 10 ng/g. Meanwhile, after 0.5 h from the injection of 12.5 mg/kg (i.p.) of scoparone, we observed an average concentration of around 20 ng/g in brain tissue which was four times higher than with the lower dose. Thus, the two times higher dose gave a four times higher concentration in the healthy brain. According to the results from the PA assay, the procognitive effect was higher for the lower dose (5 mg/kg) of scoparone in both acute and subchronic treatment regiments, suggesting the existence of high-affinity molecular targets for scoparone (see below). After acute treatment, the higher dose of scoparone (12.5 mg/kg) caused a twofold lower procognitive effect than the lower dose (5 mg/kg). Thus, it will be interesting to elucidate the precise mode of action underlying the effects on behavior and lipid remodeling within the ECS.

In conclusion, our data demonstrate that scoparone reaches the brain rather inefficiently. Since scoparone may be effluxed via p-glycoprotein and eliminated efficiently through fast peripheral metabolism, its overall brain exposure might be restricted. However, already low nanomolar concentrations of scoparone in the brain mediated significant precognitive and anxiogenic-like effects in mice, which is a remarkable finding. The potent effects of scoparone on lipid remodeling in the ECS may, at least in part, account for the differential behavioral anxiogenic effects between the acute and subchronic treatments, the latter treatment may have a significant impact on stress. There are several possible limitations of this study that relate to the complexity of the behavioral experiments, the lipidomic analyses which were only performed in the behavioral experiments, and the preliminary nature of the pharmacokinetic data that require confirmation in fully dialyzed mice to determine the blood–brain ratio. Despite the preliminary nature of our experiments, the findings could have translational implications. Given its apparent potency, scoparone may contribute to the alleged psychoactive effects of liquors containing *Artemisia* species containing high amounts of this coumarin as shown for *A. umbelliformis*. In future studies, we aim to address the mode of action of scoparone and related coumarins on lipid remodeling and its overall effect on the central ECS.

## Experimental procedures

### Plant material and isolation procedure

Scoparone was isolated from *Artemisia umbelliformis* Lam. (*Asteraceae*) collected and authenticated in summer 2013 in the Medicinal Plant Garden, Medical University, Lublin, Poland. Dried aerial parts (40 g) were subjected to extraction with methanol under the reflux for 90 min. A sample of dried crude extract (200 mg) was used for the isolation of scoparone. For the isolation, the hydrodynamic Spectrum HPCCC (Dynamic Extractions, Slough, UK) multilayer coil-planet J-type centrifuge, equipped with either two analytical coils connected in series (22 ml total volume) or two preparative coils (137 ml total volume), was used. HPCCC separation was performed on a two-phase solvent system composed of n-heptane, ethyl acetate, methanol, and water with the volume ratio of 2:3:2:3, giving the *Kval* for target compound equal to 0.96. A scale-up process was developed from the analytical to the preparative stage. 35 mg of scoparone was obtained with a purity > 98% (HPLC).

### Animals

Swiss albino mice (total number 372 mice) were housed 4 per cage group in the 12 h light/dark cycles with free access to food and water (room temperature 21 ± 1 °C). Each experimental group was comprised of 9–10 animals randomly allocated to prevent bias. 10-weeks old male Swiss albino mice (35–40 g) were used for the LPS model of cognitive impairment in the PA test. 6-weeks old male Swiss mice (25–30 g) were used for all other experiments, except the FST in which both males and females were tested for sex differences in response to the scoparone treatment.

### Ethics

All experiments were performed according to the permission of the Local Ethics Committee (95/2019, 44/2020, 45/2020, 1/2021). The experiment was in accordance with the National Institute of Health Guidelines for the Care and Use of Laboratory Animals and approved by the European Community Council Directive for the Care and Use of Laboratory Animals of 22 September 2010 (2010/63/EU). Authors complied with the ARRIVE guidelines. Simple randomisation method and blinding during the outcome assessment, the data analysis, during allocation, and the conduct of the experiment.

### Materials and treatment

0.9% sterile sodium chloride solution for injections (0.9% NaCl) was used to prepare solutions of scopolamine, borneol, LPS, and imipramine (Sigma-Aldrich, St. Louis, MO, USA). Solutions of scoparone were prepared by suspension in one drop of 1% Tween 80 solution and gradually dissolving in the saline. Scoparone was obtained as described above. Injections (i.p.) were made at the volume of 10 ml/kg of mice body weight, and fresh solutions were prepared every day before administration. The scopolamine LPS and imipramine doses were chosen based on literature data^39–41^ and our previous studies^14^. The doses of scoparone (2.5, 5, 12.5, 15, 25 mg/kg) were chosen based on our pilot study and literature data^42^. 15 mg/kg of scoparone was used in the LPS-model of memory impairment (PA test) based on our previous study^43^. The doses of borneol (50 mg/kg) were chosen based and our preliminary study. Control groups were injected with 0.9% sterile sodium chloride solution for injections (0.9% NaCl) each time. In the acute model of administration, mice received a single scoparone injection 30 min before the tests (PA, FST, EPM). In the extended subchronic experiments, scoparone was administered once a day for 14 days at 9 a.m. (FST, EPM, and PA tests). On the 15th day of the treatment, 30 min after scoparone injection, a behavioral test was performed. LPS- and scopolamine-induced memory deficits, as well as borneol treatment (48 mice), were described below and in the Supplementary Information.

### Behavioral tests and neuropharmacological assessment

#### Elevated plus maze (EPM)

The EPM was performed following the procedure described previously^44^. The overall time that mice (80 mice) spent in open arms were measured using timers. The number of entrances to enclosed and open arms of the maze was tested after single (40 mice) and subchronic (40 mice) administration of scoparone (2.5, 5, 12.5, 25 mg/kg).

#### Passive avoidance test (PA)

The acquisition of memory processes was evaluated in the PA test (88 mice) using the same procedure described previously^14,45^.

For the scopolamine-induced model of memory impairment, scoparone (5 and 12.5 mg/kg) was administered 30 min before the pre-test, and 10 min after coumarin injection, mice (48 mice) were treated with scopolamine (1 mg/kg, i.p.), or saline in the control group.

The model of LPS memory impairment was established experimentally by our group^43^ (40 mice). Scoparone (15 mg/kg, i.p.), or saline in the control group were administered 60 min after a single injection of LPS (0.8 mg/kg), and then only coumarin was injected once daily for 6 consecutive days. On the seventh day, scoparone or saline was administered once (9.00 a.m.), 30 min before the first trial (memory acquisition) and re-tested after 24 h.

### LC–MS/MS measurements and quantification of coumarins

#### Sample storage and extraction

Immediately after the FST (Supplementary Information) and EPM tested mice were decapitated, the whole brain tissues were collected, rinsed in the 0.9% saline solution for injections from male mice, and stored at − 80 °C until further LC–MS/MS and biochemical analyses. Specimens were mechanically homogenized (three steel beads) in acetonitrile (1:4 w/v). Homogenate was centrifuged at 13,000 rpm, 10 min., at + 4 °C. 50 µl of supernatant was spiked with 5 µl of IS solution (25 ng/ml of psoralen). A 10 µl of aliquot was injected into the LC–MS/MS system for analysis.

### LC–MS/MS analytics and quantification of coumarins in the brain

A hybrid triple quadrupole API 4000 QTRA*P*(Sciex) was used with Shimadzu UHPLC. Chromatography was performed on a Synergi™ 4 µm Fusion-R*P*80 Å C18, 50 × 2 mm (Phenomenex). The column temperature was maintained at 45 °C, and subsequently, a gradient of 0.1% (v/v) formic acid in water (solvent A) and 0.1% formic acid in methanol (solvent B) was used as follows: gradient of 95% A to 5% A during 0.2–4.2 min, then linear 95% A at 4.40–5.00. The flow rate was 0.8 ml/min, and the autosampler was cooled at + 4 °C.

The MS detector was operated in the positive electrospray ionization (ESI) mode using multiple reaction monitoring (MRM) transitions (Supplementary Table S1). There were used the ion spray voltage (4,000 V), ion spray temperature (450 °C), curtain gas (30 psi, GS1 65 psi, and GS2 35 psi). Data were acquired and processed using Analyst software. Supplementary Fig. S1 shows *m/z* in QI ESI scans. Calibration curves in the biological matrix were generated by plotting the peak area ratio (y) of all coumarins to IS versus its nominal concentration (x) using a weighted (1/ × 2) linear regression model. A coefficient of correlation (r ≥ 0.9900) was obtained for all compounds. In the applied method, scopoletin and isoscopoletin were not distinguished. To estimate the elimination kinetics of scoparone from the brain the software PCModfit Version 6 was used, assuming negligible background from brain vasculature and that a constant fraction of the substance in the brain is eliminated per unit time.

### Preparation of the calibration standard solution

Scoparone stock solutions (1 mg/ml in methanol) were prepared and used for further dilutions. All the solutions were stored at 4 °C. A calibration curve in the range 0.02–1000 ng/ml was prepared by spiking a 5 µl aliquot of standard solutions with 45 µl blank tissue supernatant and 5 µl of IS solution (25 ng/ml). A 10 µl of aliquot was then injected into the LC–MS/MS system for analysis. Recovery of analytes from brain matrix was assessed from different independent experiments as > 95% for scoparone, > 92% for scopoletin, > 95% for isoscopoletin, and > 91% for isofraxidin.

### Biochemical studies

#### Determination of AChE and BChE levels in brain homogenate

The intact brain without the cerebellum, dissected immediately after behavioral tests, was homogenized in ice-cold PBS (10% w/v) and centrifuged at 10,000 rpm at 4 °C for 15 min. The supernatant was collected and stored at − 80 °C for further analysis of AChE and BChE. The PierceTM BCA Protein Assay Kit determined protein concentration.

For AChE and BChE determination, the ELISA kit (Cloud-Clone Corp., USA) was used, and all measurements were done according to the manufacturer’s protocol. Absorbance was read at 450 nm, and obtained data were presented as pg/mg protein.

#### FRAP evaluation in brain homogenate

The FRAP assay was performed using a protocol for a 96-well plate^46^. Freshly prepared FRAP working solution 10:1:1 (300 mM acetate buffer, pH 3.6; 10 mM TPTZ in 40 mM HCl; 20 mM FeCl_3_) was added to the brain homogenate samples (see above). After 20 min of incubation at 37 °C, absorbance at 593 nm was read. Calculations were made in relation to the ascorbic acid standard curve and the absorbance of the blank sample (PBS). The antioxidant capacity was expressed as µM Ascorbic Acid Equivalent (AAE) per mg of protein.

#### Activity-based protein profiling (ABPP)

The ABPP was chosen to investigate possible effects on the activity of serine hydrolases in the mouse brain. ABPP experiments were performed by using membrane preparations obtained from mouse brains as described by Ref.^47^, with minor modifications. In brief, brain samples (25 µg of total membrane proteins) were incubated with the serine hydrolase targeting probe FP-TAMRA (ActivX™) at a final concentration of 500 nM and incubated for 15 min at RT. To visualize the possible modulation of the activity of the crucial lipid-metabolizing serine hydrolases that regulate endocannabinoid degradation (FAAH; MAGL, ABHD6, and ABHD12), brain samples were preincubated with scoparone and the positive controls (all from Cayman Chemical, UK), namely URB597 (1 µM); JZL184 (1 µM); WWL70 (10 µM); THL (10 µM) or DMSO (as vehicle control) for 30 min at 37 °C. After applying the FP-TAMRA probe for 15 min, the incubation the reaction was stopped by 4 × Lämmli Buffer, and the samples were boiled at 70 °C for 10 min. Samples were cooled down to RT and loaded into an SDS-PAGE gel as was previously described^47^.

#### Targeted LC–MS/MS lipidomics

Endocannabinoid levels were measured in the brain tissue as described recently^48^ in an attempt to explain the scoparone-induced anxiogenic behavior in mice. Tested in FST and EPM mice were euthanized by decapitation, and the brain was collected immediately, hemispheres were separated and rinsed in cold PBS and snap frozen in liquid N_2_. Samples were stored at − 80 °C until extraction. A previously described protocol was used to extract the lipids^29^. 10 μl of the final sample were injected into the column for LC–MS/MS quantification. The analysis was conducted as described previously^29^ except for using a Shimadzu UFLC coupled to a TripleQuad 5500 QTRAP mass spectrometer (AB Sciex, Canada). AEA and 2-AG were measured using the Turbo-Ion Spray interface operated in positive mode. Reprosil-PUR LC C18 column (3 μm particle size; 2 × 50 mm; Dr. A. Maisch HPLC GmbH, Germany) maintained at 40 °C was used. The mobile phase [mixture of (A) 2 mM ammonium acetate plus 0.1% formic acid and (B) methanol plus 2 mM ammonium acetate] at the flow rate of 0.35 ml/min was applied. The MS parameters of the ESI source were as follows: curtain gas, 30 psig; Ion Spray voltage, 4.5 kV; temperature, 600 °C; ion source gas, 50 psig.

Prostaglandin E2 and D2 were measured using the Turbo-Ion Spray interface operated in negative mode. The same column (Reprosil-PUR C18 column; 3 μm particle size; 2 × 50 mm) maintained at 40 °C with a mobile phase flow rate of 0.30 ml/min was used with the mixture of (A) 2 mM ammonium acetate plus 0.1% formic acid and (B2) acetonitrile plus 0.1% formic acid as mobile phase. The MS parameters of the ESI source were as follows: curtain gas, 30 psig; Ion Spray voltage, 4.5 kV; temperature, 600 °C; ion source gas, 50 psig. Gradient elution was used starting with 95% phase A and a linear increase of phase B2 reaching 40% at 3 min. Then the linear increase rate was decreased to reach 65% B2 at 9 min. Another linear increase of phase B2 was employed to reach 95% at 10 min and maintained for 4 min. Finally, phase B2 was linearly decreased to 5% at 15 min and maintained at this concentration for 2 min for re-equilibration. The total analysis time was 17.0 min. The following MRM transitions were monitored: PGE2, *m/z* 351.1 → 189.0 (PGE2-d4 355.0 → 319.0), PGD2 351.1 → 189.0 (PGE2-d4 355.0 → 319.0).

#### Statistics

For statistical analysis GraphPad Prism 8.3.1 was used. When appropriate, one or two- way ANOVA analyses were performed for statistical analysis with post hoc Bonferroni’s or Tukey’s test. All data are shown as means (± SEM). The results of *p* < 0.05 were considered statistically significant. Outliers were calculated using Grubbs' test, alpha = 0.05. The index of latency (IL) was calculated to evaluate changes in memory acquisition and consolidation. IL = (TL2 − TL1)/TL1 where TL1 was the time to enter the dark compartment during the training and TL2 was the time to re-enter the dark compartment during the retention.

## Supplementary Information

Supplementary Information.

## Data Availability

All data generated or analyzed during this study are available from the corresponding author on reasonable request.
