# Supplementary material for: Neuropsychopharmacological profiling of scoparone in mice
Source: Sci Rep. 2022 Jan 17;12:822. doi: 10.1038/s41598-021-04741-3 (PMC8764054; doi:10.1038/s41598-021-04741-3)
Supplement: Supplementary file 1 — Supplementary Information. [file 41598_2021_4741_MOESM1_ESM.docx]

***LC-MS/MS analytics and quantification of coumarins in the brain***


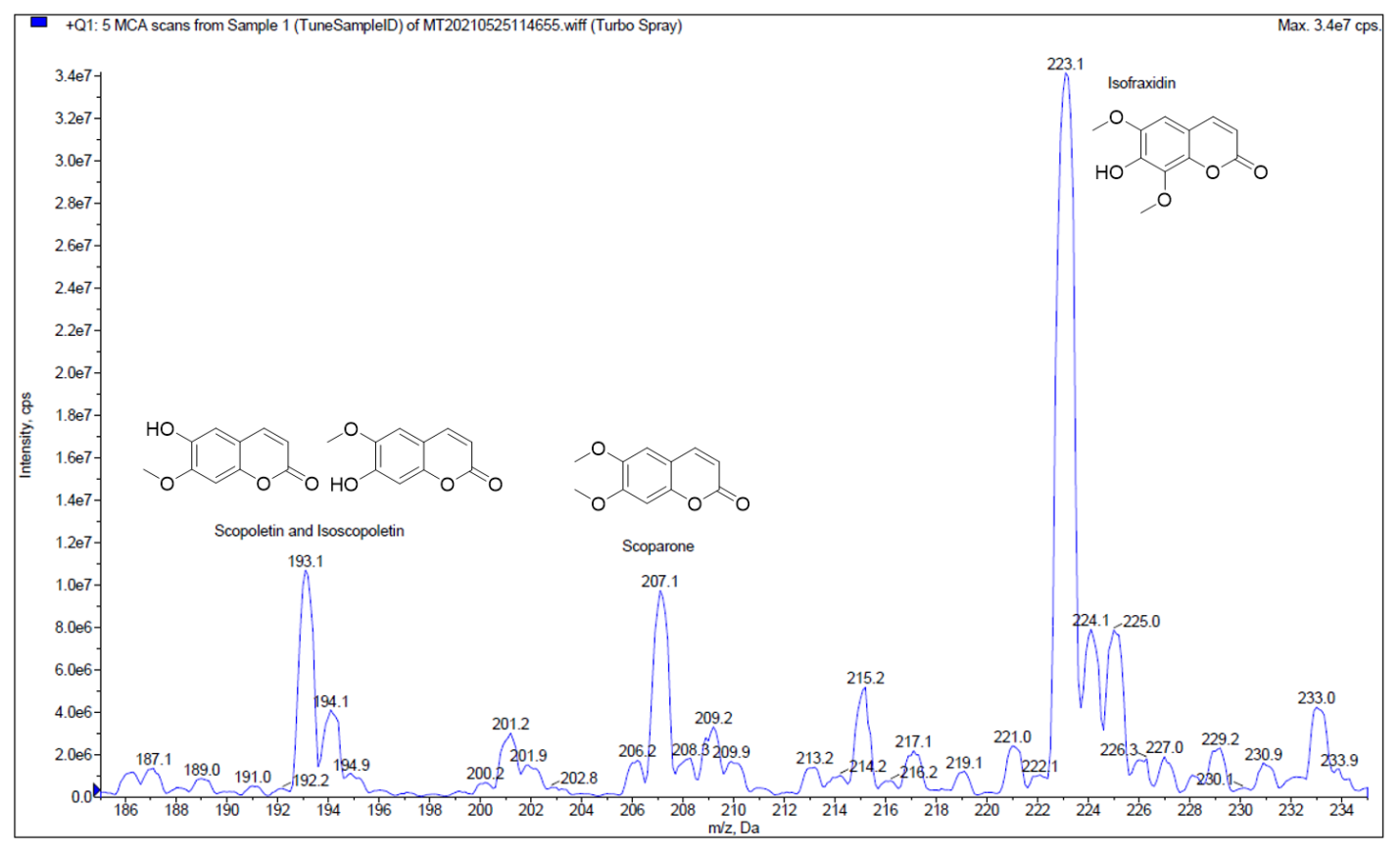


**Figure S1.** Exemplary QI ESI scans of coumarins analyzed.

**Table S1.** Multiple reaction monitoring (MRMs) condition for **1** and internal standard (psoralen)

| Analyte | Retention time {min) | Mode | Precurson ion (m/z) | Product ion (m/z) | Declustering potential (eV) | Collision energy (eV) |
| --- | --- | --- | --- | --- | --- | --- |
| Scoparone | 1.82 | Positive | 207.1 | 151.1 | 70 | 29 |
|  |  |  | 207.1 | 107.1 | 70 | 49 |
| Psoralen (IS) | 2.02 | Positive | 187.2 | 131.1 | 105 | 32 |

***Effect of scoparone treatment on stress and depressive-like behavior in the FST in female and male mice***

**Forced Swim Test (FST).** The FST (Porsolt test) was performed as described previously ^1^. A horizontally situated camera recorded the total time of the assay. The measurement of the immobility time was carried out using the Kinoscope Open Source program ^2^. Both male (52 mice) and female (52 mice) mice were tested to evaluate sex differences 30 min after a single administration of scoparone (2.5, 5, 12.5, 25 mg/kg), and only males were tested for subchronic administration of scoparone at the same doses.

Our primary aim was to profile possible neuropharmacological effects of scoparone in diverse behavioral paradigms and therefore we limited *i.p.* injections to maximum four doses (2.5, 5, 12.5, and 25 mg/kg).

As summarized in Supplementary Table S2, the effect of scoparone in the FST was measured in the Kinoscope Open-Source program as the immobility time. A two-way ANOVA analysis revealed statistically significant effects for the treatment of the positive control drug imipramine (F (5, 104) = 19.11, *p* <0.0001) with no effect for sex (F (1, 104) = 0.020, *p* = 0.8877) or sex treatment interaction (F (5, 104) = 1.24, *p* = 0.3046). Data obtained with the positive control imipramine validated the FST model (p < 0.05 male and p < 0.001 female). However, scoparone (2.5, 5, 12.5, and 25 mg/kg) had no significant effect on immobility time in either female or male mice. We also studied the effect of subchronic administration of scoparone (14 days of daily injections) in the FST. As show in Supplementary Table S2, imipramine administered subchronically decreased immobility time (p < 0.0001) while scoparone (2.5, 5, 12.5, and 25 mg/kg) had no significant effect on the observed parameter.

**Table S2.** Effect of scoparone (2.5, 5, 12.5, and 25 mg/kg, *i.p*) and imipramine (IMI, 30 mg/kg *i.p.*) administered acutely on depressive-like behaviors in the FST in male and female Swiss albino mice. Animals were injected with scoparone 30 min before the test (see methods). Data show mean values ± SD, n=8-9, post hoc Bonferroni’s test.

|  | Saline | IMI 30 mg/kg | Scoparone  2.5 mg/kg | Scoparone  5 mg/kg | Scoparone  12.5 mg/kg | Scoparone  25 mg/kg |
| --- | --- | --- | --- | --- | --- | --- |
| male | 174.10 ±  8.08 | 104.4 ±  59.07  * | 137.89 ±  3.852 | 161.76 ±  17.59 | 200.26 ±  15.29 | 181.58 ±  11.94 |
| female | 186.59 ±  7.35 | 66.68 ±  37.51  *** | 155.49  ±  12.04 | 157.51 ±  14.90 | 192.96 ±  12.84 | 194.21 ±  11.55 |

***Effect of subchronic scoparone treatment on stress and depressive-like behavior in the FST in mice***

As summarized in Supplementary Table S3, the effect of subchronic administration of scoparone in the FST was measured in the Kinoscope Open-Source program as the immobility time. A one-way ANOVA analysis revealed statistically significant effects (F (5, 52) = 9.842; *p* < 0.0001). Indeed, imipramine administered subchronically decreased immobility time (p < 0.0001). However, scoparone (2.5, 5, 12.5, and 25 mg/kg) had no significant effect on the observed parameter (52 mice).

**Table S3.** Effect of scoparone (2.5, 5, 12.5, and 25 mg/kg, *i.p.*) and imipramine (IMI, 15 mg/kg *i.p.*) administered subchronically (14 days) on depressive-like behaviors in the FST in Swiss albino male mice. Data show mean values ± SD, n=8-9, post hoc Tukey’s test.

|  |  |
| --- | --- |

|  | Saline | IMI 15 mg/kg | Scoparone  2.5 mg/kg | Scoparone  5 mg/kg | Scoparone  12.5 mg/kg | Scoparone  25 mg/kg | F, p |
| --- | --- | --- | --- | --- | --- | --- | --- |
| male | 161.4±  14.59 | 48.57±  13.91  *** | 167.3±  15.17 | 186.1±  13.21 | 162.2±  21.32 | 156.5±  10.67 | F (5, 52) = 9.842; *p* < 0.0001 |

***Effect of subchronic scoparone treatment on anxiety-like behavior in the EPM in mice***

In Supplementary Table S4 effects of subchronic administration of scoparone in the EPM test is shown. A one-way ANOVA analysis did not reveal statistically significant influence on anxiety level assessed as a percentage of open arm entries and time spend in the open arms (40 mice).

**Table S4.** Effect of chronic administration of scoparone (2.5, 5, 12.5, and 25 mg/kg, *i.p* 14 days) on anxiety level assessed as a percentage of open arm entries and time spend in the open arms in the EPM paradigm in Swiss albino male mice. Data show mean values ± SD, n=8.

|  | Vehicle  (saline) | Scoparone  2.5 mg/kg | | Scoparone  5 mg/kg | | | Scoparone 12.5 mg/kg | Scoparone  25 mg/kg | F, p |
| --- | --- | --- | --- | --- | --- | --- | --- | --- | --- |
| percentage of open arms entries  (mean ± SEM) | 10.52±  2.894 | 9.208±  3.137 | | 9.998±  3.367 | | | 8.929±  3.388 | 5.404±  2.009 | F (4, 40) = 0.5580  *p* = 0.6946 |
| percentage of open arms time  (mean ± SEM) | 2.215±  0.7017 | | 1.950±  0.8913 | | 1.027±  0.4772 | 2.016±  1.286 | | 1.402±  1.235 | F (4, 40) = 0.2834  *p* = 0.8866 |

***Effect of acute scoparone treatment on locomotor activity in the EPM in mice***

The percentage of open arm entries was decreased after administration of scoparone at the doses of 5 and 25 mg/kg (p < 0.05 and p < 0.01, respectively), while rather unexpectedly and the dose of 12.5 mg/kg of scoparone did not influence this behavior [F (4, 40) = 0.8009, p = 0.5320]. Scoparone at 2.5 mg/kg only moderately decreased locomotor behavior (Supplementary Table S5).

**Table S5.** Effect of acute administration of scoparone (*i.p.* 30 min before test) on locomotor activity assessed as a total number of arm entries in the EPM paradigm in Swiss albino male mice.

|  | Vehicle  (saline) | Scoparone  2.5 mg/kg | Scoparone  5 mg/kg | Scoparone  12.5 mg/kg | Scoparone  25 mg/kg |
| --- | --- | --- | --- | --- | --- |
| total number of arms entries  (mean ± SEM) | 13.56 ±  1.44 | 10.80 ±  1.32 | 13.14 ±  1.34 | 11.38 ±  1.05 | 11.80 ±  1.28 |

***Effects of borneol on memory improvement induced by acute administration of scoparone in PA test***

The acquisition of memory processes was evaluated in the PA test using the same procedure described previously ^3,4^.

Borneol was administered 60 min before scoparone (5 and 12.5 mg/kg, *i.p.*). The first trial was performed 30 min after scoparone injection and animals were re-tested 24 hours after the last injection.

Supplementary Fig. S2 shows effects of acute administration of borneol (50 mg/kg *i.p.*) on scoparone (5 and 12.5 mg/kg, *i.p.*) induced memory acquisition improvement using the PA test in mice (48 mice) (two way ANOVA: borneol pre-treatment (n=8 [F (1, 8) = 8.183; *p* < 0.01) and scoparone treatment (F (2, 24) = 13.39, *p* < 0.0001) and interactions [F (2, 8) = 6.201; *p* < 0.0236). The post hoc Bonferroni’s test confirmed that scoparone given acutely significantly increased LI value (5 mg/kg - *p* < 0.001; 12.5 mg/kg - *p* < 0.05) as observed previously. Borneol did not affect scoparone induced memory improvement.

**Figure S2.** Effects of acute administration of borneol (BOR, 50 mg/kg *i.p.*) on scoparone (SCOP, 5 and 12.5 mg/kg, *i.p.*) induced memory acquisition improvement using the PA test in mice. Borneol was administered 60 min before scoparone (5 and 12.5 mg/kg, *i.p.*). The first trial was performed 30 min after scoparone injection and animals were re-tested 24 hours after the last injection; n = 8; the means ± SEM; * *p* < 0.05; *** *p* < 0.001 vs. vehicle-treated control group; Bonferroni’s post hoc test.


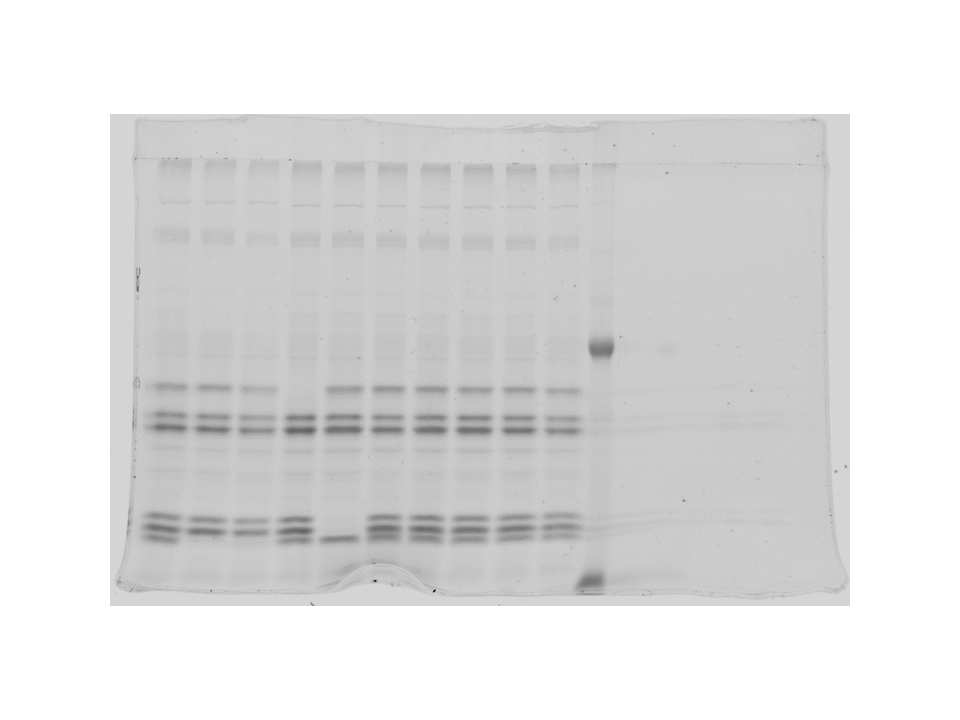


Full unprocessed gel for activity-based protein profiling (related to Figure 8)

### **References**

1. Porsolt, R. D., Anton, G., Blavet, N. & Jalfre, M. Behavioural despair in rats: A new model sensitive to antidepressant treatments. *Eur. J. Pharmacol.* **47**, 379–391 (1978).

2. Kokras, N., Baltas, D., Theocharis, F. & Dalla, C. Kinoscope: An Open-Source Computer Program for Behavioral Pharmacologists. *Front. Behav. Neurosci.* **11**, 1–7 (2017).

3. Michalak, A., Pekala, K., Budzynska, B., Kruk-Slomka, M. & Biala, G. The role of verapamil and SL-327 in morphine- and ethanol-induced state-dependent and cross state-dependent memory. *Eur. J. Pharmacol.* **834**, 318–326 (2018).

4. Skalicka-Wozniak, K., Budzynska, B., Biala, G. & Boguszewska-Czubara, A. Scopolamine-Induced Memory Impairment Is Alleviated by Xanthotoxin: Role of Acetylcholinesterase and Oxidative Stress Processes. *ACS Chem. Neurosci.* **9**, 1184–1194 (2018).
